# First Complete Genome Sequences of Genogroup VI Porcine Sapoviruses

**DOI:** 10.1128/genomeA.00275-14

**Published:** 2014-04-03

**Authors:** Tomoichiro Oka, Linda J. Saif, Zongzhao Yang, Kelly A. Scheuer, Garrett T. Stoltzfus, Qiuhong Wang

**Affiliations:** a Food Animal Health Research Program, Ohio Agricultural Research and Development Center, Department of Veterinary Preventive Medicine, College of Veterinary Medicine, the Ohio State University, Wooster, Ohio, USA; b Department of Virology II, National Institute of Infectious Diseases, Tokyo, Japan; c Hangzhou Bureau of Animal Husbandry and Veterinary Medicine, Hangzhou, China

## Abstract

Sapoviruses, members of the family *Caliciviridae*, are genetically diverse and divided into multiple genogroups. Only a few complete genome sequences of animal strains are available. We report the first complete genome sequences of genogroup VI sapoviruses, those of strains JJ674 and JJ681, isolated from fecal samples from diarrheic pigs.

## GENOME ANNOUNCEMENT

Sapoviruses (SaVs), members of the family *Caliciviridae*, have been isolated from humans, swine, mink, dogs, sea lions, and bats. The SaV genomes are divided into 14 genogroups (genogroup I [GI] to GXIV) based on the capsid sequences (1). Currently, complete genomes are available only for GI to GV, GVII, and GXIV SaVs. We determined the full-length genome sequences of two GVI SaVs, Po/SaV/GVI/OH-JJ681/2000/US (JJ681) and Po/SaV/GVI/OH-JJ674/2000/US (JJ674), isolated from feces from two diarrheic postweaning pigs from the same Ohio farm on 22 December 2000 (2, 3). The 3′-end 3-kb sequence of JJ681 has been reported previously (2). The upstream region (1.5 kb) of JJ681 was amplified by nested reverse transcription (RT)-PCR using random hexamers (Invitrogen) and gene-specific reverse primers with Z-Taq DNA polymerase (TaKaRa) and GoTaq DNA polymerase (Promega). Then, the region further upstream (1.5 kb) was amplified using a forward primer, designed based on an SaV conserved motif corresponding to nucleotides (nt) 1,408 to 1,421 of the Po/SaV/GIII/Cowden strain (GenBank accession no. AF182760) (4) and a specific reverse primer using EX-Taq DNA polymerase (TaKaRa). The 5′-end 1.4-kb region was determined by the 5′-RACE method. Briefly, homopolymeric (dC or dA) tailing and nested PCR were performed with gene-specific primers and the abridged anchor primer (AAP) (5′-GGCCACGCGTCGACTAGTACGGGIIGGGIIGGGIIG-3′) and the abridged universal primer (AUAP) (5′-GGCCACGCGTCGACTAGTAC-3′) (Invitrogen) for poly(C)-tailed cDNA and primers QT (5′-CCAGTGAGCAGAGTGACGAGGACTCGAGCTCAAGCTTTTTTTTTTTTTTTTT-3′) (5) and QO (5′-CCAGTGAGCAGAGTGACG-3′) (5) for poly(A)-tailed cDNA. Each PCR product was sequenced after being cloned into a pCR4 Topo vector (Invitrogen). To confirm JJ681 sequences, three overlapping PCR fragments (0.6 kb, 2.4 kb, and 2.7kb) covering the 5′-end genomic region, including the 5′ untranslated region (5′-UTR) and most of open reading frame 1 (ORF1), were amplified with PrimeStar HS DNA polymerase (TaKaRa) and sequenced following cloning into a pCR4 Blunt-Topo vector (Invitrogen). We also analyzed the entire genome of the JJ674 strain using primer sets designed based on the JJ681 strain and PrimeStar HS DNA polymerase. The six overlapping PCR fragments (0.3 kb, 1.4 kb, 2.0 kb, 1.7 kb, 1.7 kb, and 3.4 kb) were sequenced directly.

The full-length genome sequence was assembled and analyzed by the Sequencher version 4.10.1 program (GeneCodes) and Genetyx Mac version 16.0.4 software (Genetyx Corporation).

The genomes of JJ681 and JJ674 consist of 7,198 nt, excluding the poly(A) tail. The genomes were predicted to contain two ORFs: nucleotide positions 11 to 6,667 (ORF1) and 6,664 to 7,170 (ORF2). The predicted 5′- and 3′-UTRs were 10 nt and 28 nt long, respectively. A single amino acid difference between the JJ681 and JJ674 strains was predicted in ORF2.

Based on the predicted ORF1 amino acid sequences, the newly determined GVI SaVs are closest (53%) to the Po/SaV/GVII/K7 strain (GenBank accession no. AB221130) (6).

The accumulation of complete genome sequences of multiple genogroups of SaVs is essential to establish classification schemes based on both nonstructural and structural regions to assess virus recombination, to design broadly reactive or genogroup-specific reverse transcription (RT)-PCR assays, and to identify the key structural and functional amino acid residues in viral protein(s).

### Nucleotide sequence accession numbers.

The genome sequences of JJ674 and JJ681 have been deposited in GenBank under the accession numbers KJ508818 and AY974192, respectively.
